# A multicenter international prospective study of the validity and reliability of a COVID-19-specific health-related quality of life questionnaire

**DOI:** 10.1007/s11136-022-03272-2

**Published:** 2022-10-23

**Authors:** Cecilie Delphin Amdal, Ragnhild Sørum Falk, Susanne Singer, Madeline Pe, Claire Piccinin, Andrew Bottomley, Lambert Tetteh Appiah, Juan Ignacio Arraras, Oliver Bayer, Eirik Alnes Buanes, Anne Sophie Darlington, Gracia Dekanic Arbanas, Kristin Hofsø, Bernard Holzner, Pernilla Sahlstrand-Johnson, Dagmara Kuliś, Ghansyam Parmar, Niveen M. E. Abu Rmeileh, Melanie Schranz, Samantha Sodergren, Kristin Bjordal

**Affiliations:** 1 Research Support Services, Oslo University Hospital, Oslo, Norway; 2 Department of Oncology, Oslo University Hospital, Sogn Arena, Post Box 4950 Nydalen, NO-0424 Oslo, Norway; 3 Institute of Medical Biostatistics, Epidemiology and Informatics, University Medical Centre of Johannes Gutenberg University Mainz, Mainz, Germany; 4 Quality of Life Department, European Organisation for Research and Treatment of Cancer, Brussels, Belgium; 5 Komfo Anokye Teaching Hospital, Kumasi, Ghana; 6 Servicio de Navarro de Salud, Pamplona, Spain; 7 Department of Anaesthesia and Intensive Care, Haukeland University Hospital, Bergen, Norway; 8 Norwegian Intensive Care and Pandemic Registry, Haukeland University Hospital, Bergen, Norway; 9 School of Health Sciences, University of Southampton, Southampton, UK; 10 Clinical Hospital Centre Rijeka, Rijeka, Croatia; 11 Division of Emergencies and Critical Care, Department of Research and Development, Oslo University Hospital, Oslo, Norway; 12 Lovisenberg Diaconal University College, Oslo, Norway; 13 University Hospital for Psychiatry I, Medical University of Innsbruck, Innsbruck, Austria; 14 Department of Otorhinolaryngology, Skåne University Hospital, Lund, Sweden; 15 Department of Pharmacy, Sumandeep Vidyapeeth Deemed to be University, Vadodara, India; 16 Institute of Community and Public Health, Birzeit University, Birzeit, Palestine; 17 Faculty of Medicine, University of Oslo, Oslo, Norway

**Keywords:** COVID-19, Quality of life, Patient-reported outcome measure, Questionnaire, PROM, HRQoL

## Abstract

**Purpose:**

To develop and validate a health-related quality of life (HRQoL) questionnaire for patients with current or previous coronavirus disease (COVID-19) in an international setting.

**Methods:**

This multicenter international methodology study followed standardized guidelines for a four-phase questionnaire development. Here, we report on the pretesting and validation of our international questionnaire. Adults with current or previous COVID-19, in institutions or at home were eligible. In the pretesting, 54 participants completed the questionnaire followed by interviews to identify administration problems and evaluate content validity. Thereafter, 371 participants completed the revised questionnaire and a debriefing form to allow preliminary psychometric analysis. Validity and reliability were assessed (correlation-based methods, Cronbach’s α, and intra-class correlation coefficient).

**Results:**

Eleven countries within and outside Europe enrolled patients. From the pretesting, 71 of the 80 original items fulfilled the criteria for item-retention. Most participants (80%) completed the revised 71-item questionnaire within 15 min, on paper (*n* = 175) or digitally (*n* = 196). The final questionnaire included 61 items that fulfilled criteria for item retention or were important to subgroups. Item-scale correlations were > 0.7 for all but nine items. Internal consistency (range 0.68–0.92) and test–retest results (all but one scale > 0.7) were acceptable. The instrument consists of 15 multi-item scales and six single items.

**Conclusion:**

The Oslo COVID-19 QLQ-W61© is an international, stand-alone, multidimensional HRQoL questionnaire that can assess the symptoms, functioning, and overall quality of life in COVID-19 patients. It is available for use in research and clinical practice. Further psychometric validation in larger patient samples will be performed.

**Supplementary Information:**

The online version contains supplementary material available at 10.1007/s11136-022-03272-2.

## Plain English summary

Many people have been affected by COVID-19, with an impact on their health-related quality of life. Health-related quality of life includes physical, emotional, and social elements. It is important that we can ask the right questions to assess quality of life after having had COVID-19. Our work focuses on testing the questionnaire we have developed, to make sure it includes the right questions, and that patients understand these. We asked patients from eleven countries to complete the questionnaire. A total of 425 patients completed the questionnaire. Sixty one of 80 questions were kept, and the wording on several items was changed. The final 61-item questionnaire includes the right questions, is complete, and is acceptable to patients. This questionnaire is valid and can be used with patients during and after COVID-19 to measure their health-related quality of life.

## Introduction

For over two years, the SARS-CoV-2 virus has continued its worldwide spread causing a heavy burden for the many affected by symptomatic disease (COVID-19). Vaccination programs have reduced the risk of severe disease, hospitalization, death [1–3], and disease transmission [4]. Fully vaccinated individuals still experience symptoms ranging from mild to serious [5], especially if they are older (≥ 65 years) and have comorbidities [6]. The most frequent symptoms reported in COVID-19 are fever, headache, cough, myalgia, dyspnea, and loss of taste and smell [7–9]. They may also suffer from, e.g. sore throat, runny nose, gastrointestinal problems, and chest pain [7–10]which can have a considerable impact on physical, emotional, and social functioning [11, 12]. In April 2020, we conducted a literature review and identified publications related to studies of symptoms and problems with functioning in patients with COVID-19 from a large variety of clinical settings, culture and countries, and from all continents. As the pandemic continued to evolve with new variants of the virus, we updated our literature review twice during the first year [9].

Given the impact of COVID-19 and the potential for persisting problems, it is crucial to evaluate the patient’s perspective using validated patient-reported outcome measures (PROMs). Such measures can be used to assess symptoms and other relevant health-related quality of life (HRQoL) issues over time and in response to therapeutic interventions. They help ensure that the patient’s perspective remains central and mitigate the effects of underreporting by health-care professionals [13, 14].

To date, there is no cross-culturally validated COVID-19-specific HRQoL questionnaire available. Although several COVID-19-related clinical outcome assessments have been published [15], they focus on the pandemic’s impact on the general population [16, 17] and mental health [18] or describe more general recommendations [19]. Generic HRQoL measures, along with symptom checklists, have been employed to assess issues in patients with COVID-19 [20, 21], but they may not capture the range of potential symptoms and HRQoL issues associated with COVID-19. Moreover, ad hoc non-COVID-specific measures may fail to demonstrate the psychometric properties required for a PROM [22].

In April 2020, our multilingual and multicultural research group, involving clinicians, psychometricians, statisticians, and HRQoL specialists, set out to develop a questionnaire to assess HRQoL and symptoms in patients with COVID-19, from diagnosis through active disease and recovery. The first two phases of the questionnaire development have been published [9, 23]. In this paper, we present the next phase of the development process with pretesting of our provisional COVID-19 questionnaire and preliminary psychometric testing of the validity and reliability in an international sample of patients.

## Methods

The current study is a multicenter international methodology study for a questionnaire development. Guidelines from the European Organisation for Research and Treatment of Cancer (EORTC) Quality of Life Group (QLG) were followed [24]. The four-phase procedure covers general principles for questionnaire development and is supported by the Food and Drug Administration [25]. The first two phases (gathering relevant issues through literature review, interviews with health-care workers and patients in seven countries, and operationalization from issues to items) were performed from April to October 2020 and have been published [9, 23].

To make sure that the questionnaire also covered potential new important issues evolving during the pandemic the literature review was updated in October 2020, with results that was further explored in this phase III study, and in February 2021 without any new issues being reported [9].

The resulting 80-item provisional weekly (PW) questionnaire was named the OSLO COVID-19 QLQ-PW80© (short name QLQ-PW80) and copyrighted by the Oslo University Hospital, Norway that coordinated the international development. In the current phase III study, this work continued with further pretesting (pilot testing in a small sample) and validation of the provisional questionnaire. The aim was to identify missing or redundant items and ensure comprehensibility (phase IIIA) and perform initial psychometric testing (phase IIIB). All study documents were developed in English. The questionnaire was translated from English into the required languages (*n* = 9) using a modified forward/backward translation procedure based on international guidelines [26] and reviewed by an experienced translation officer before the patient enrollment started.

### Countries and participants in phase IIIA and phase IIIB

Countries from all continents were approached through professional networks, including the World Health Organization (WHO) COVID-19 clinical management team network. Partners from 11 countries (Austria, Croatia, Germany, Ghana, India, Norway, Palestine, Spain, Sweden, The Philippines, and United Kingdom) enrolled patients ensuring representation from different cultural areas.

The target population was ≥ 18 years, with verified SARS-CoV-2 infection (according to local/national standards) and active or previous symptomatic COVID-19. Patients in hospitals, nursing homes, at home, or in COVID-19 centers were eligible if they were able to read and comprehend the study documents. Patients in intensive care units could be enrolled after discharge. To ensure content validity of the final questionnaire according to guidelines, pre-specified enrollment matrices were used [24]*.*These matrices were set up to ensure sufficient sample sizes with adequate distribution of participants to represent the target population. In phase IIIA (Online Appendix 1), we aimed to include 10–15 patients in each cell of the sample matrix, ≥ 5 patients per country, and ≥ 45 patients in total. In phase IIIB (Online Appendix 2), we aimed to include 15 patients per cell of the sample matrix and ≥ 300 patients in total [24], to allow for preliminary evaluation of the psychometric properties, in particular the hypothesized scale structure. In phase IIIB, participants in recovery, expected to have stable disease i.e., unlikely to experience any changes in the physical well-being, were asked to complete the questionnaire a second time after 14 days (± 2 days) to measure test–retest reliability. The target sample size for this part was 50 patients.

### Phase IIIA: procedure, collection of data

Data collection in phase IIIA was performed from November 2020 until June 2021. The researchers in the 11 countries approached patients in hospital, nursing homes, and patients staying at home. After written informed consent, patients completed the provisional questionnaire, the QLQ-PW80, in their native language and recorded the completion time. This was followed by structured debriefing interviews by the research team documented with field notes (Online Appendix 3). Patients’ background information was collected (age, gender, hospitalization, time since diagnosis, disease severity, comorbidity). Whether the patients had experienced each item was assessed considering the whole disease period and reported as *relevance* (1 = not at all, 2 = a little, 3 = quite a bit, 4 = very much). If experienced, the extent they were troubled by this was regarded as a *measure of importance* (0 = not experienced, 1 = not at all to 4 = very much). We defined relevant items as those with *relevance* scores 2–4, and important items as those with *importance* scores 3 and 4. Patients were asked if any of the items were difficult to understand or confusing, annoying, or upsetting, and if any of the items overlapped with other items. The interviewer also asked about relevance and importance of any other issues not listed, but still experienced by the patient. Six items were assessed in more detail to explore how the patients interpreted/understood these issues (Appendix 4).

### Phase IIIA: decision rules and criteria for retention of items

A set of six decision rules and criteria for retention of items in phase IIIA was set up as recommended [24]; the first four criteria considered most important (Table 1A). Since COVID-19 is a new disease and a limited number of patients were to be enrolled in phase IIIA, care was taken not to exclude important items simply because of low prevalence. The decision on whether an item should be retained, modified, or removed was made by consensus in the project group, including review of potentially overlapping items and rewording of difficult and annoying items.

**Table 1:** Criteria for retention of items in the Oslo COVID-19 QLQ phase IIIA and IIIB

A: Phase IIIA, The Oslo COVID-19 QLQ-PW80
Items that < 10 participants have experienced (relevance score 2–4), if one or more of them found it important
Items where ≥ 10 participants scored relevance 2–4, and at least 25% found them important
< 5% of the participants found the item difficult
< 5% of the participants found the item upsetting
Mean score > 1.5
No floor or ceiling effect, > 10% responses in category 3&4 or 1&2
B: Phase IIIB, The OSLO COVID-19 QLQ-PW71
Mean relevance score > 1.5
Importance score 3&4 > 50%
No floor or ceiling effect, > 10% responses in category 3&4 or 1&2
Use of the entire range of the Likert-scale (score 1–4)
< 5% of the participants found the item difficult
< 5% of the participants found the item upsetting
The proportion of patients that completed the item > 95%

### Phase IIIB: procedure and collection of data

Data collection in phase IIIB was performed over six months from July 2021 to January 2022. An electronic data capture system, Ledidi® was used that fulfilled the necessary national and EU security and privacy policy requirements [27]. After written informed consent, patients in hospital, at home or in nursing home, were asked to complete questionnaires on paper or digitally in the Ledidi system®. The set of questionnaires consisted of (1) background information (as described for phase IIIA, supplemented by vaccination status), (2) the provisional questionnaire resulting from phase IIIA, and (3) a debriefing questionnaire. The debriefing questionnaire documented the time and help needed to complete the questionnaire, whether there were difficult, annoying or overlapping items, or additional issues not covered by the questionnaire. In addition, patients in recovery (more than three months after being infected) were asked to point out any additional issues they had experienced more than three months after being infected.

### Phase IIIB: decision rules and criteria for retention of items

In phase IIIB, the research group used seven criteria for retention to decide on items that were candidates for removal (Table 1B). The summarized results and consistency across languages were discussed, and decision trails from earlier phases of development consulted. An item was kept if clinically relevant, fulfilling ≥ 5 of the retention criteria, or found important to subgroups of patients, and not overlapping with other items. An item was modified if > 5% of the patients found it difficult or annoying.

### Statistical analyses

#### Descriptive analyses

Descriptive analyses were presented as frequencies and proportions for categorical data and means, standard deviations, and range for continuous data. For the analysis of content validity, interviews in phase I and pilot testing by the user group in phase II laid the foundations [23]. We investigated this further by exploring feasibility; i.e., patients who needed more than > 30 min to complete the questionnaire, patients who needed help understand the items, and whether there were systematic patterns of missing values. We explored possible differences in response patterns between countries by qualitative review of responses and comments in the debriefing questionnaire. New issues raised by the patients were considered if not covered by existing items, not excluded in previous phases, and not clinical parameters or other distinct conditions.

#### Psychometric analyses

The scale structure analyses were based on the COnsensus-based Standards for the selection of health Measurement INstruments (COSMIN) taxonomy [28]. In addition to the descriptive analyses, we tabulated the results per item along with the number of fulfilled criteria (Online Appendix 5). The multi-language clinical expert group proposed scales from a clinical point of view. The scale structure was modified based on the following analyses. We assessed the validity by correlation-based methods (e.g., multitrait analysis) using the Pearson correlation. Item convergent validity was supported by a correlation of ≥ 0.40 between an item and its own scale (corrected for overlap). Scales that are conceptually related (e.g., fatigue and malaise) are expected to have a correlation ≥|0.40| while scales that are conceptually different (e.g., worries and temperature) would have a correlation <|0.40|. Scaling errors were calculated as the percentage of items that correlated higher with a different scale than with their own scale, corrected for overlap.

Known group comparisons were performed for pre-defined groups (Online Appendix 6) based on the patient matrix (Online Appendix 2), using independent sample *t* tests.

To test the reliability, we calculated the internal consistency using Cronbach’s alpha for each multi-item scale. Values > 0.70 were considered acceptable for group comparisons. Test–retest reliability was assessed by calculating intra-class correlation coefficients (ICC) for scores at first and second time points for the multi-item scales.

Confirmatory factor analyses were performed to explore the dimensionality of the questionnaire. We calculated standardized factor loadings for each item with regard to the corresponding scale and considered loadings > 0.40 to be sufficient. Model fit was assessed by the Comparative Fit Index (CFI) and the Tucker-Lewis Index (TLI), with both indices considered to indicate good fit if > 0.95 as well as by the Root-Mean-Squared Error of Approximation (RMSEA) which is recommended to be < 0.06 [29].

The final scale structure was based on the psychometric tests and clinical evaluation, and a scoring manual was developed (Online Appendix 7). All items had responses on a four-point Likert scale ranging from 1 = ‘not at all’ to 4 = ‘very much,’ except the two items in the overall quality of life scale ranging from 1 = ‘very poor’ to 7 = ‘excellent.’ The scores from scales and single items were linearly transformed to scores ranging from 0 to 100. A higher score represented worse symptoms and more problems on functioning scales. For the overall quality of life scale, higher score represented better quality of life.

## Results

### Phase IIIA

**Fig. 1:**
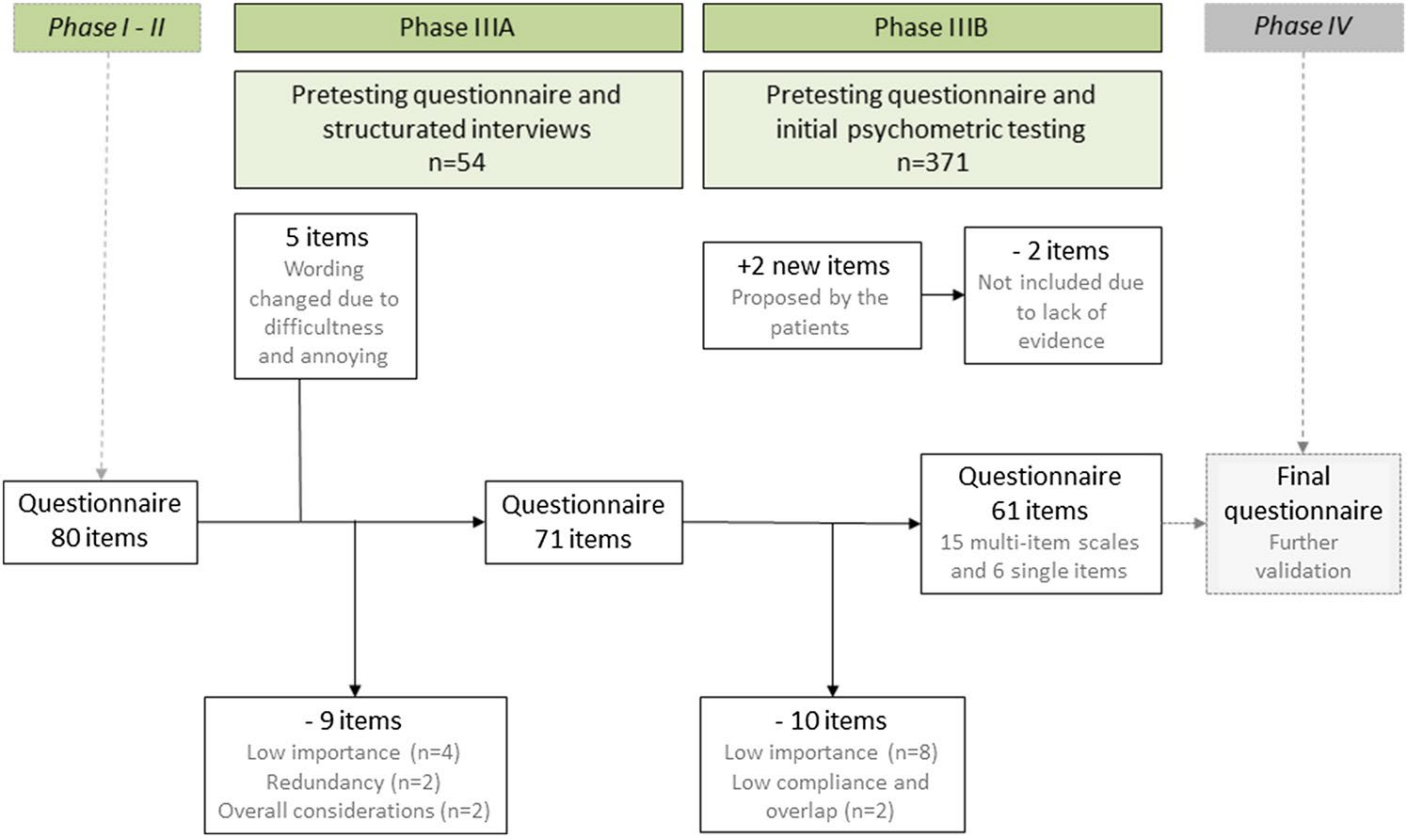
Overview of the development of the international COVID-19 specific health-related quality of life questionnaire

Research teams in 11 countries interviewed 54 patients (Table 2). Adult males and females of all ages, with active ongoing disease or in recovery, admitted to hospital or isolated at home, were included. Twenty-one patients reported various comorbidities. The mean time needed to complete the QLQ-PW80 was 16 min (range 4–45). The patient-reported *relevance* and *importance*, and item score distribution with final decisions, are presented in Online Appendix 8. In summary, 21 items were candidates for removal according to criteria outlined in Table 1A. Patients identified possible overlap for five groups of items. Nine items were removed (Fig. 1) due to low importance (*n* = 4, blocked nose, sneezing, shaking hands, heartburn), overlap (*n* = 2, abdominal discomfort, abandoned by family and friends), and an overall consideration with low mean score, floor effect, limited importance, and few patients experiencing them (*n* = 3, constipation, dysuria, and hair loss). Five items identified as difficult or annoying were reworded, resulting in the revised provisional 71-item questionnaire named the Oslo Covid-19 QLQ-PW71© (QLQ-PW71).

**Table 2:** Patient characteristics in phase IIIA and IIIB

Patient characteristics	Phase IIIA *n* = 54	Phase IIIB *n* = 371
**Age**		
Mean (range), years	46.2 (21–88)	47.2 (18–110)
**Age groups**	n (%)	n (%)
18–40 years	27 (50)	152 (41)
41–69 years	21 (39)	173 (47)
≥ 70 years	6 (11)	46 (12)
**Gender**		
Female	32 (59)	207 (56)
Male	22 (41)	164 (44)
**Country–Language**		
Austria–German	5 (9)	16 (4)
Croatia–Croatian	5 (9)	30 (8)
Germany–German	5 (9)	46 (12)
Ghana–Twi	5 (9)	15 (4)
Ghana–English		28 (8)
India–Gujarati	5 (9)	45 (12)
Norway–Norwegian	5 (9)	44 (12)
Palestine–Arabic	5 (9)	39 (11)
Spain–Spanish	5 (9)	40 (11)
Sweden–Swedish	5 (9)	37 (10)
The Philippines–Filipino	5 (9)	
United Kingdom–English	4 (7)	31 (8)
**Hospitalization**		
At home	32 (59)	263 (71)
In hospital	22 (41)	90 (24)
In nursing home or other institution	–	17 (5)
Unspecified	–	1 (0.3)
**Disease status**		
Shortly after diagnosis (up to 7 days after diagnosis)	7 (13)	80 (22)
During active disease in institution or at home	18 (33)	
Subacute (< 14 days after discharge/four weeks after diagnosis)	9 (17)	53 (14)
Four weeks to 3 months after diagnosis	20 (37)	56 (15)
More than three months after diagnosis	–	181 (49)
Unspecified	–	1 (0.3)
**WHO clinical severity of disease**		
Mild/Moderate –did not need/receive oxygen	40 (74)	284 (77)
Severe –received oxygen	11 (20)	62 (17)
Critical –received invasive ventilation	3 (6)	24 (6)
**Co-morbidity (Charlson)**		
No	32 (59)	236 (64)
Yes	21 (39)	135 (36)
Unknown	1 (2)	
Type of co-morbidity^a^		
Chronic cardiac disease	2 (4)	15 (4)
Chronic kidney disease	1 (2)	5 (1)
Chronic liver disease	3 (6)	2 (0.5)
Chronic neurologic disorder	2 (4)	3 (0.8)
Chronic pulmonary disease	4 (7)	12 (3)
Diabetes	2 (4)	31 (8)
Hypertension	9 (18)	50 (13)
Immunodeficiency	2 (4)	
Malignant neoplasm	1 (2)	3 (0.8)
Mental disorder	1 (2)	10 (3)
Other, not specified	7 (14)	49 (13)
**Vaccination (partially or fully) against COVID-19**	Not assessed	
Yes, before		115 (31)
Yes, after		113 (30)
No		49 (13)
Do not wish to answer		3 (1)
Missing^b^		91 (25)

Numbers are presented as frequencies and proportions if not otherwise specified. Numbers may not add up due to rounding

^a^Multiple responses possible

^b^The missing of vaccination status was due to inclusion of this variable after recruitment had started

### Phase IIIB

A total of 371 patients from 10 countries with ongoing disease or in recovery, admitted to hospital or isolated at home were interviewed (Table 2, Online Appendix 9). Patients who reported unspecified comorbidity (*n* = 49) commented, e.g., that they had slow metabolism, lumbar prolapse, fibromyalgia, Crohn’s disease, and myalgic encephalomyelitis. The six patients who needed > 30 min to complete the questionnaire (Table 3) were elderly (67–106 years old) and needed assistance with completion. Forty-four patients needed help to understand the items, mainly from Ghana (*n* = 15), India (*n* = 8), and Germany (*n* = 7), all age groups were represented. Patients in hospital (27/90) and nursing home/other institution (3/17) needed assistance more often compared to patients at home (14/263). Even though they needed assistance, 63% completed the questionnaire within 15 min.

**Table 3:** Phase IIIB Feasibility of the OSLO COVID-19 QLQ-PW71 in 371 patients

	n (%)
Time needed to complete the questionnaire, minutes	
≤ 10	189 (51)
11–15	109 (29)
16–30	49 (13)
> 30	6 (2)
Missing	18 (5)
Help with completion	
No	271 (73)
Yes, read and/or write	38 (10)
Yes, understand	25 (7)
Yes, both understand and read and/or write	19 (5)
Missing	18 (5)
Type of questionnaire	
Paper version	175 (47)
Digital version	196 (53)

Most patients (279, 75%) completed all 71 items. Of the 92 patients with missing values, 68 had only one or two missing. Seven of the 92 patients had completed only the first page of the digital questionnaire (item 1–23), missing the subsequent 48 items. For the others, there were no patterns of missing data. There were no clear differences between the countries regarding number and pattern of missing values.

### Additional patient-reported problems

Some patients described additional symptoms or problems experienced in the first three months (*n* = 56) and/or more than three months after being diagnosed (*n* = 36). Two of these problems were new: menstrual disturbances (*n* = 2) and word-finding problems or aphasia (*n* = 2). Other proposed issues were either covered by the questionnaire (*n* = 34), excluded previously in phase I or phase IIIA (*n* = 11), or were not patient-reported symptoms but objective signs such as low oxygen saturation and weight loss (*n* = 11) (Online Appendix 10). The project group agreed that there was not enough evidence to include the two new issues.

### Evaluation of items and (initial) scale structure

Of the 71 items, 55 items fulfilled ≥ 5/7 criteria for retention (Online Appendix 5). Twenty items had a mean score > 1.5. Across all items, the whole range of responses was used and patients did not find them difficult or annoying in any language. Compliance was high (> 95%) for all but two items (light and heavy housework). All items were reviewed quantitatively and qualitatively. It was decided to remove 10 items that did not fulfill more than 4/7 criteria for retention, and were either not important to subgroups of patients (*n* = 8) or had low compliance and overlapped with other items (*n* = 2). For the initial proposed scales, the internal consistency was satisfactory (Cronbach’s alpha > 0.70) for all but one scale (Sensory) (Online Appendix 5). Nevertheless, the scale was retained because it was considered clinically meaningful.

For the initial proposed scales, the item-scale correlations were satisfactory for all but nine items (drowsy, feeling ill or unwell, headache, chest pain, weakness in hands or feet, appetite loss, carrying a heavy bag upstairs, social activity, worry about financial difficulties). Three were moved to another scale (drowsy, headache, chest pain) while six were kept in the initial scale, as this was considered clinically more meaningful. Three items (palpitation, burning and sore eyes, skin problems) were kept as single items. The ‘Respiratory’ scale was divided into ‘upper’ (throat) and ‘lower’ (chest), as this was clinically more meaningful, and the item-scale correlations were virtually unchanged.

### The validity and reliability of the final questionnaire

The final 61-item weekly questionnaire resulting from our current phase IIIB development, now consists of 15 multi-item scales and six single items and was named the Oslo COVID-19 QLQ-W61© (QLQ-W61) (Table 4). All scales had acceptable item convergent validity (|≥ 0.40|) except one item (financial difficulties) in the ‘Worries’ scale. The scaling error was low (0–3.3%). Discrimination across pre-defined groups was observed for most of the scales (Temperature disruption, Sensory, Gastrointestinal, Physical and Social functioning), but not for all (Pain, Worries, Emotional, and Cognitive functioning) (Table 5). The ‘Respiratory lower, chest’ scale reached significance for disease status, but not for comorbidity and gender.

**Table 4:** Reliability and scale structure of the Oslo COVID-19 QLQ-W61

Scale	Acronym	No. of items	Mean score^a^	Cronbach’s Alpha	Test–retest with ICC (95% CI)	Scaling error %
Temperature disruption	TP	2	22.9	0.85	0.83 (0.68; 0.90)	0.0
Fatigue	FA	3	49.1	0.92	0.86 (0.74; 0.92)	1.6
Sleep	SL	1	30.8	n.a	0.85 (0.73; 0.92)	1.6
Malaise	MA	4	32.2	0.83	0.83 (0.69; 0.91)	1.6
Pain	PA	5	26.5	0.84	0.92 (0.84; 0.95)	1.6
Respiratory lower, chest	RL	5	22.3	0.87	0.92 (0.85; 0.95)	3.3
Respiratory upper, throat	RU	3	16.4	0.68	0.85 (0.73; 0.92)	0.0
Palpitations	PP	1	17.6	n.a	0.95 (0.91; 0.97)	1.6
Eye	EY	1	11.7	n.a	0.44 (− 0.08; 0.71)	0.0
Sensory	SE	2	29.4	0.91	0.88 (0.77; 0.94)	0.0
Neurological	NE	2	17.7	0.73	0.90 (0.80; 0.95)	0.0
Appetite loss	AP	1	25.1	n.a	0.80 (0.61; 0.89)	0.0
Gastrointestinal	GI	3	14.3	0.78	0.91 (0.82; 0.95)	0.0
Skin	SK	1	11.5	n.a	0.72 (0.48; 0.85)	0.0
Emotional functioning	EF	8	20.0	0.88	0.93 (0.86; 0.96)	3.3
Cognitive functioning	CF	3	15.6	0.73	0.95 (0.91; 0.97)	0.0
Physical functioning	PF	3	17.1	0.79	0.96 (0.92; 0.98)	0.0
Role functioning	RF	1	30.0	n.a	0.90 (0.81; 0.95)	1.6
Social functioning	SF	2	24.3	0.74	0.85 (0.71; 0.92)	0.0
Worries	WO	8	21.3	0.78	0.90 (0.74; 0.96)	0.0
Overall quality of life	QOL	2	35.1	0.91	0.92 (0.85; 0.96)	0.0

^a^Range 0–100 where a higher score represents worse symptoms and more problems on functioning scales. For the overall quality of life scale, higher score represents better quality of life

*CI* confidence interval; *ICC* intra-class correlation coefficient

**Table 5:** Known group validity; Comparisons of scales within the Oslo COVID-19 QLQ-W61 for clinically distinct groups

Scales	Patient groups		Difference	
			Mean (95% CI)	p-value
	Young patients (≤ 40 years)	Elderly patients (> 70 years)		
Cognitive functioning	15.9	14.5	1.4 (-5.3; 8.1)	0.68
Gastrointestinal	17.6	8.5	9.1 ( 1.3; 16.9)	0.02
Sensory	34.4	13.6	20.8 (7.9; 33.7)	0.002
	Acute disease (shortly after diagnosis and during active disease)	Recovery (more than three months after diagnosis)		
Temperature	29.8	15.6	14.3 (8.2; 20.3)	< 0.001
Respiratory lower, chest	27.8	16.6	11.1 (6.2; 16.1)	< 0.001
Pain	26.3	26.7	− 0.4 (-5.7; 4.9)	0.88
Sensory	37.6	20.9	16.7 (9.2, 24.2)	< 0.001
Social functioning	31.5	16.6	15.0 (8.9; 21.0)	< 0.001
Worries	22.9	19.7	3.2 (-0.6; 7.0)	0.10
	Co-morbidity Yes	Co-morbidity No		
Respiratory lower, chest	25.4	20.6	4.8 (-0.4; 10.0)	0.07
Physical functioning	26.8	11.5	15.3 (10.3; 20.4)	< 0.001
	Female	Male		
Respiratory lower, chest	22.4	22.2	0.1 (-5.0; 5.2)	0.96
Emotional functioning	21.2	17.9	3.3 (-1.2; 7.7)	0.15

*CI* confidence interval

Cronbach’s alpha was > 0.70 for 14 of the 15 multi-item scales (range 0.68–0.92) (Table 4). The test–retest reliability was good, ICCs between the two time-points were > 0.70 except for problems with eyes (ICC 0.44). In the confirmatory factor analysis, all standardized factor loadings exceeded the threshold of 0.40 (range 0.42–0.95), supporting the hypothesized scale structure. The CFI was 0.85, the TLI was 0.83, and the RMSEA was 0.06, somewhat lower than the pre-defined values of acceptable fit of the model and the data.

## Discussion

This questionnaire was developed to capture HRQoL of patients with COVID-19 from diagnosis through active disease and the recovery period. We have demonstrated that the questionnaire captured relevant and important issues to the international sample of COVID-19 patients, that the final number of items is manageable, and that the instrument shows promising psychometric properties.

To allow the questionnaire to be feasible and valid in a heterogeneous international setting, we successfully ensured that patients involved represented a good distribution of age, gender, disease phases and severity, comorbidities, and countries. Cross-cultural acceptability was supported by patients from 11 countries in three continents reporting the items to be relevant and important, and discussions among the involved researchers from different cultural areas. Special attention was given to the wording of five items pointed out as difficult to understand to improve cross-cultural acceptance. The word ‘stigmatised’ was difficult to understand in some languages and the group decided to include ‘or judged negatively’ to improve the comprehension of the concept (Online Appendix 8, item 71). The word ‘abandoned’ was described as too offensive in some languages and, based on earlier input from patients, this was changed to ‘not receive sufficient attention’ (Online Appendix 8, item 77).

Even though elderly patients might need some assistance, the high compliance, few missing values, and the finding that most patients used ≤ 15 min to complete the questionnaire, supported that it is easy to understand and fill in, making it suitable for clinical studies but also for descriptive purposes in clinical practice. Since the digital version of the questionnaire was challenging for some patients, more focused instructions and user-friendly layout are recommended in future studies.

Our results where the participants regarded the functional and overall HRQoL items as relevant and important, are supported by others studies using, e.g., EQ-5D [30]. It is evident that the HRQoL issues relevant to COVID-19 patients are not limited to symptoms alone; how COVID-19 has affected these patients’ functioning and overall quality of life remain important factors in assessing the patients’ experiences.

The preliminary psychometric properties of the QLQ-W61 were robust. All multi-item scales had good internal consistency. Results from *known group comparisons* supported the pre-defined hypotheses for the majority of the scales, but not for all. For ‘Pain’ and ‘Worries,’ no differences were found between patients with acute disease and those in recovery; both groups showed elevated levels. This suggests that pain (e.g., muscle aches and pain) and worries (e.g., worries about health) may be issues that need to be flagged for post-COVID-19 condition (Long COVID). Younger and elderly patients had similar results on ‘Cognitive functioning.’ This may be a result of selection bias where elderly patients who could respond to the COVID-19 questionnaire (reflecting less problems with cognitive functioning) were selected for this study. For ‘Emotional functioning,’ an expected gender difference was not found even though many studies report more emotional issues among females compared to males [31, 32]. This imply that the emotional stress of having COVID-19 may have a similar impact on both genders, but this has to be further explored in larger patient populations. As expected, patients with acute disease had higher mean score on the ‘Respiratory lower, chest’ scale (e.g., shortness of breath, chest pain) than those in recovery. Males and patients with comorbidities are at risk of having more severe COVID-19 [33, 34] and lower respiratory symptoms such as shortness of breath and chest pain are regarded as symptoms of severe disease. However, we were surprised to find that there were no differences regarding gender or comorbidities. The *test–retest reliability* for both multi-item and single item scales were of acceptable levels, except for the eye-item. This shows that the assessment is stable over time unless clinical changes occur in the well-being of the patient. Although results from the *initial confirmatory factor analysis* showed sub-optimal fit (TLI < 0.90, CFI < 0.90), this may be explained by the low sample size relative to the complexity of the scale structure for this questionnaire. Furthermore, testing of differences between countries will be performed in the next phase of the questionnaire development involving larger samples of patients from finalized clinical studies in COVID-19 patients.

One limitation might be the questionnaire’s ability to cover issues from new variants of COVID-19, and issues related to Long COVID, although we performed two updates of the literature review to reduce this risk. For example, with the Omicron variant, the most frequent symptoms were runny nose, headache, fatigue, sneezing, and sore throat, which were different from the dominant symptoms in the earlier COVID-19 Alpha variant (i.e., fever, cough, and loss of sense of smell or taste) [35]. Also, symptoms of dry and red eyes have been mentioned as more frequent with Omicron than earlier variants of the virus [36]. The QLQ-W61 captures most but not all Omicron symptoms. Runny nose was excluded after phase I, and sneezing after phase IIIA, as patients who had experienced these symptoms did not regard them as important.

In addition, due to the time constrains, there are limitations in the analyses of the questionnaire’s ability to capture all symptoms of Long COVID. Long COVID was unknown at the start of the pandemic and consequently not included in the first two phases of this project. To compensate for this in the current phase, we specifically asked the 181 patients in recovery (more than three months post infection) to point out any additional issues they had experienced more than three months after being infected. The two new issues that were proposed were not included. Menstrual disturbances were more likely to be related to vaccination [37] or secondary to psychological distress [38]. Word-finding problems could be related to general fatigue or be part of a cerebrovascular incidence secondary to COVID-19 related thrombotic complications [39]. In a study on breast cancer patients with Long COVID not yet published (JIA, personal communication), the participants completed the QLQ-PW80 7 or 10 months after being diagnosed with COVID-19. They were asked to describe any new issues, but none were reported. Long COVID has been shown to affect patients in all age groups, with varying background characteristics and levels of disease severity [40].The most commonly seen symptoms are fatigue, reduced physical and cognitive functioning, shortness of breath, and palpitations [41], but also psychological symptoms such as anxiety and depression. Other symptoms reported are loss of taste and smell, muscle pain, headache, skin problems, and hair loss [41]. Almost all symptoms of Long COVID-19 are covered in the QLQ-W61, except less common symptoms such as hair loss (removed in phase IIIA) and conditions such as post-traumatic stress disorder.

Since this process included a broad sample of international patients in various stages of COVID-19, including recovery, we believe the questionnaire fits the needs of various stakeholders. However, a wider distribution of participants would have been optimal in a study of a worldwide pandemic. More countries were approached, but due to ethical approval obstacles, unfortunately, some colleagues were not able to participate and this is a limitation. Some patients involved in this study had comorbidities or pre-existing conditions, and we cannot ascertain that all reported issues are caused by COVID-19. However, since issues were reported as relevant and important by a number of patients, it is ensured that the reported issues are common to at least a subgroup of COVID-19 patients, regardless of their pre-existing conditions.

The Oslo COVID QLQ-W61 is regarded to have the properties needed in clinical trials as a standardized internationally developed tool to evaluate HRQoL of patients during active disease, in the recovery phase, and even in a Long COVID setting. It is a comprehensible instrument that is easy to fill in, and we believe that it could be useful in clinical practice as well. In a clinical setting, the questionnaire could be used to monitor patients, e.g., for symptom control in the clinic and to evaluate whether their HRQoL issues are changing over time. This has to be explored in future studies.

Additional psychometric testing in a large international sample would be preferable, but is considered time consuming and resource intensive and may be difficult to perform. Therefore, further validation will be based on data from ongoing clinical studies where patients fill in this COVID questionnaire, and data from two such studies (not yet published) are already available. In this study, we tested the questionnaire with a weekly (W) time frame (QLQ-W61), but in future studies, we may test the daily version (QLQ-D61).

## Conclusion

The Oslo COVID QLQ-W61© is a stand-alone, multidimensional HRQoL questionnaire that can assess the symptoms, functioning, and overall quality of life of COVID-19 patients. The questionnaire is applicable for clinical trials and clinical practice, covers relevant COVID-19 issues and is acceptable to a broad population of COVID-19 patients from many countries. Although the questionnaire still needs to go through a final development phase of international psychometric validation in a large patient sample, this provisional questionnaire is now available for use with nine completed translations.

## Supplementary Information

Below is the link to the electronic supplementary material.

Supplementary file1 (DOCX 64 kb)

Supplementary file2 (XLS 78 kb)

Supplementary file3 (DOCX 45 kb)

## Abbreviations

CFI: Comparative fit index
COSMIN: COnsensus-based Standards for the selection of health Measurement INstruments
COVID-19: Coronavirus disease
EQ-5D: EuroQoL questionnaire 5 dimensions
EORTC: European Organisation for Research and Treatment of Cancer
HRQoL: Health-related quality of life
ICC: Intra-class Correlation Coefficients
Ledidi®: Software company for digital data capturing
Long COVID: Post-COVID-19 condition
PROMs: Patient-reported outcome measures
PW: Provisional Weekly
QLG: Quality of Life Group
QLQ-PW80: The OSLO COVID-19 QLQ-PW80©
QLQ-PW71: The Oslo Covid-19 QLQ-PW71©
QLQ-W61: The Oslo COVID-19 QLQ-W61©
RMSEA: Root-mean-squared error of approximation
The Oslo Covid-19 QLQ-PW71©: The revised 71-item provisional weekly questionnaire
The Oslo COVID-19 QLQ-W61©: The final 61-item weekly questionnaire
The OSLO COVID-19 QLQ-PW80©: 80-Item provisional weekly questionnaire
TLI: Tucker-lewis index
WHO: World health organization
