# Supplementary material for: A multicenter international prospective study of the validity and reliability of a COVID-19-specific health-related quality of life questionnaire
Source: Qual Life Res. 2022 Oct 23;32(2):447–59. doi: 10.1007/s11136-022-03272-2 (PMC9589865; doi:10.1007/s11136-022-03272-2)
Supplement: Supplementary file 1 — Supplementary file1 (DOCX 64 kb) [file 11136_2022_3272_MOESM1_ESM.docx]

## Appendix 1: Selection matrix for patients in phase IIIA

|  | **Phase IIIA** |
| --- | --- |
| **Number of patients** | **Total ¤** |
| **Age** |  |
| 18-40 **years** | ≥ 10 |
| 41-70 **years** | ≥ 10 |
| ≥ 71 **years** | ≥ 10 |
| **Gender** |  |
| Female | ≥ 15 |
| Male | ≥ 15 |
| **Hospitalisation** |  |
| In hospital/ At nursing home | ≥ 15 |
| At home | ≥ 15 |
| **Disease status** |  |
| Shortly after diagnosis (up to 7 days after diagnosis) and/or during active disease in institution or at home | ≥ 15 |
| Subacute (up to 14 days after discharge or four weeks after diagnosis) or late/in recovery (more than 14 days after discharge or four weeks after diagnosis) | ≥ 15 |
| **Co-morbidity** |  |
| 0 | ≥ 10 |
| ≥ 1 | ≥ 10 |
| **Language groups** |  |
| English | ≥ 5 |
| West-Germanic | ≥ 5 |
| Scandinavian | ≥ 5 |
| Romance | ≥ 5 |
| Non-European languages | ≥ 5 |
| **Total** | **≥ 45** |

## Appendix 2: Selection matrix for phase IIIB

## A minimum of 15 patients in each cell below, patients could be represented in more than one cell. Each country must recruit patients in most cells.

|  | **Language group** | | | | | | |
| --- | --- | --- | --- | --- | --- | --- | --- |
|  | English speaking | West-Germanic | Scandinavian | Slavic and Other European | Romance | Non-European languages | **Total ¤** |
| **Number of patients** | **≥ 40** | **≥ 40** | **≥ 40** | **≥ 20** | **≥ 40** | **≥ 60** | **300** |
| **Countries** | **UK, Ghana** | **Germany, Austria** | **Norway**  **Sweden** | **Croatia** | **Spain** | **India**  **Palestine**  **Ghana-Twi** |  |
| **Age** |  |  |  |  |  |  |  |
| 18-40 years |  |  |  |  |  |  |  |
| 41-70 years |  |  |  |  |  |  |  |
| ≥ 71 years |  |  |  |  |  |  |  |
| **Gender** |  |  |  |  |  |  |  |
| Female |  |  |  |  |  |  |  |
| Male |  |  |  |  |  |  |  |
| **Hospitalisation** |  |  |  |  |  |  |  |
| In hospital/ At nursing home |  |  |  |  |  |  |  |
| At home |  |  |  |  |  |  |  |
| **Disease status** |  |  |  |  |  |  |  |
| Shortly after diagnosis (up to 7 days after diagnosis) and/or during active disease in institution or at home |  |  |  |  |  |  |  |
| Subacute (up to 14 days after discharge or four weeks after diagnosis) or late/in recovery (more than 14 days after discharge or four weeks after diagnosis) |  |  |  |  |  |  |  |
| **Co-morbidity** |  |  |  |  |  |  |  |
| 0 |  |  |  |  |  |  |  |
| ≥1 |  |  |  |  |  |  |  |
| **Total** | **59** | **62** | **81** | **30** | **40** | **107** |  |

**Appendix 3:**

**Development of an international questionnaire to assess
patient-reported symptoms related to COVID-19 disease,
the OSLO COVID-19 QLQ - PW80**

**Phase IIIA - Interviews with patients with active or previous COVID-19 disease**

**Instruction to interviewer in red**

| ***Text to read aloud during interview in cursive and boxed*** |
| --- |

Please read the document in full before you start the interview.

**Documents needed for the interview**

- The OSLO COVID-19 QLQ-PW80
- CRF
- This interview guide
- The Response form (2 copies)

After the interview, please enter all data into the Ledidi platform

**Procedure for each participant**

1. Please be sure to obtain informed consent.
2. Fill in the CRF with inclusion/exclusion criteria and patient characteristics.
3. Please ask the patient to fill in the OSLO COVID-19 QLQ-PW80 and ask the patient to record the time needed for filling in the questionnaire and document it on top of the questionnaire.
4. Conduct structured interviews of the questionnaire items to investigate whether all items are clear and accepted by the participants.

| - *We are asking for your help in making a questionnaire which will be used to assess the experiences of patients who have or have had COVID-19 disease.* - *Here you can see a preliminary version of the questionnaire with 80 items that have been reported as relevant and important by other patients and health care professionals* - *Based on your experiences, could you please fill in the questionnaire?  Afterwards I will ask you about your opinion on the relevance, but also possible difficulties with each of the items.* - *Please record the time needed and document it on top of the questionnaire.* |
| --- |

**Introduction – asking the participant to fill in the questionnaire**

**Patient/participant interview procedure**

1. Please check that all items in the patient-reported questionnaire have been completed and then start the interview using **the Response form for patient interview, Table 1-3**. Be sure to respond to all questions.
   Please give a copy of the response form to the patient for reference, but the interviewer, rather than the patient should fill in the form.
2. Please be aware that we now want to explore the patient’s experience **for the whole disease period,** not only the past week. The patient may have filled in “not at all” for items that they have actually experienced previously. These items should also be explored in **Table 1**.
3. Five items need to be explored further, outlined in red in Table 1. Please provide the patients explanations.
4. Please document additional comments and comments regarding a daily version (**Table 2**) and proposals for additional items (**Table 2**)**.**

| **After the participant has completed the questionnaire**   - *Did you write down how long you needed to complete the questionnaire? Thank you for checking.*   *We will now review each item you have responded to together. Here is a copy of the form I will be filling out. . For some of the items we have an additional question.*  **For each item:**   - *Could you please tell me for each one the extent to which you have experienced it during your illness,  and to what extent you were troubled by this, as a measure of importance?*   - Response – fill in **Table 1**: - *Was the item difficult to respond to or confusing?*   - Response – fill in **Table 1** - *Was the item annoying, or upsetting?*    - Response – fill in **Table 1** - *If you think it is difficult, confusing, annoying or upsetting, how would you have asked this question?*   - Response – fill in **Table 1** - *Please tell me if you think that any of the items are overlapping or have the same meaning as other items?*   - Response – fill in **Table 1** - *If you should keep one of them, which item do you prefer?*   - Response – fill in **Table 1** - *Do you have any additional comments?*   - Response – fill in **Table 2** |
| --- |
| **Time frame:**   - *Did you notice the time frame used in the questionnaire “during the past week”?*   *We are also planning a daily version of the questionnaire with the same items included, but using the time frame “past 24 hours” and would like to hear your opinion on that (time frame, length, importance etc).*   - - Response – fill in **Table 2** |
| **Additional relevant issues**   - *Have you experienced any other issue related to COVID-19 not covered?  If so, could you please tell me for each one the extent to which you have experienced it during your illness, and to what extent you were troubled by this, as a measure of importance. Are there any overlap? Additional comments?*    - Response – fill in **Table 3** |
| **General tips for follow-up questions during the interview**  **If the answers are too general and indefinite, the follow-up may be**   - *In what way?* - *Just how do you mean?* - *Can you give me an example?*   **If the answers are incomplete, the questions may be:**   - *Any other reasons?* - *Would you tell me a little more about that?*   **Other follow-ups could ask:**   - *What makes you think this?* - *What was there about the issue that made you feel that way?* |

**Appendix 4: Questions qualitatively assessed in phase IIIA**

| **No.** | **Item** | **Question about the item** | **Summary results interviews** | **Conclusion** |
| --- | --- | --- | --- | --- |
| 13 | Have you had a feeling of tightness in your chest? | Please explain with your own words how you understand the expression “tightness in your chest”? | Patients had similar understanding of the issue. Pressure on the chest/ constriction in the chest making it difficult to breath. | The item was kept unchanged |
| 20 | Have you had feeling of tightness in your throat? | Please explain with your own words how you understand the expression “tightness in your throat”? | Patients had similar understanding of the issue. Choking feeling, suffocating or breath tightness | The item was kept unchanged |
| 33 | Have you had shooting or burning pain in your body? | Please specify which part(s) of your body that you have/had shooting or burning pain? | Patients described this type of pain in different parts of the body | The item was kept unchanged |
| 35 | Have you had weakness in your hands or feet? | The weakness; is it only in your hands and feet or is it also in your arms and legs? | Most described this in hands and feet. Some experienced it also in arms and legs. A few also described weakness in other parts of the body | The item was kept unchanged |
| 36 | Have you had tingling or numbness in your hands or feet? | The tingling or numbness; is it only in your hands or feet or is it also in your arms and legs? | Hands and feet most common | The item was kept unchanged |
|  | **Extra item:**  Has your voice sounded different as a result of your disease or treatment? | Please explain with your own words how your voice has sounded differently? (Hoarse or weak or in any other way?) | 5 patients experienced that their voice had changed. Three described weaker voice, one hoarseness. Not found important | The item was not included |
|  | **The time frame**  We are planning a daily version of the questionnaire with the same items, but using the time frame “past 24 hours” and would like to hear your opinion | Opinions about a daily version | Different opinions. Some patients misunderstood the question. | Daily version will be tested in separate study |

**Appendix 6: Phase IIIB Known group comparison of predefined groups and scales**

| **Patient characteristics** | **Patient groups** | **Scales** |
| --- | --- | --- |
| Age | Elderly patients (>70 years) vs young patients ( ≤ 40 years) | Cognitive functioning |
|  |  | Gastrointestinal |
|  |  | Sensory |
| Disease status | Acute disease (shortly after diagnosis and during active disease) vs recovery (more than three months after diagnosis) | Temperature |
|  |  | Respiratory lower |
|  |  | Pain |
|  |  | Sensory |
|  |  | Social functioning |
|  |  | Worries |
| Comorbidity | Yes vs no | Respiratory lower |
|  |  | Physical functioning |
| Gender | Female vs male | Respiratory lower |
|  |  | Emotional functioning |

**Appendix 8: Phase IIIA detailed results of criteria for removal and final decision (n = 54)**

|  | **Criteria** | **1** | **2** |  | **3** | **4** |  | **5** | **6** |  |  |  |
| --- | --- | --- | --- | --- | --- | --- | --- | --- | --- | --- | --- | --- |
| **Q** | **Item** | **Relevance score 2-4***  **Number of patients** | **Importance score 3-4****  **Number of patients (%)** | **Missing relevance/**  **important** | **Difficult**  **%** | **Upsetting**  **%** | **Observations** | **Mean score** | **% category**  **1&2 / 3&4** | **Potential overlap** | **Decision to keep or remove after phase IIIA** | **Reason** |
| 1 | Have you had fevers? | 34 | 20 (59) | 0/0 | 0 | 0 | 54 | 2.4 | 54/46 |  | Keep | Fulfil criteria |
| 2 | Have you had chills? | 27 | 14 (52) | 0/0 | 2 | 0 | 54 | 2.0 | 67/33 |  | Keep | Fulfil criteria |
| 3 | Have you needed to rest? | 49 | 30 (61) | 0/0 | 2 | 0 | 54 | 3.2 | 20/80 | Q5 | Keep | Fulfil criteria  Different concepts |
| 4 | Have you felt weak? | 48 | 30 (63) | 0/0 | 2 | 0 | 54 | 3.2 | 22/78 |  | Keep | Fulfil criteria |
| 5 | Have you been tired? | 49 | 31 (63) | 0/0 | 0 | 0 | 54 | 3.2 | 22/78 | Q6 | Keep unchanged | Fulfil criteria  Different concepts |
| 6 | Have you felt drowsy? | 44 | 25 (57) | 0/0 | 2 | 0 | 54 | 2.6 | 41/59 | Q5 | Keep unchanged | Fulfil criteria  Different concepts |
| 7 | Have you had problems sleeping? | 33 | 17 (52) | 0/0 | 2 | 0 | 54 | 2.0 | 70/30 |  | Keep | Fulfil criteria |
| 8 | Have you felt ill or unwell? | 44 | 29 (66) | 0/0 | 2 | 0 | 54 | 2.9 | 35/65 |  | Keep | Fulfil criteria |
| 9 | Have you been dizzy? | 25 | 18 (72) | 0/0 | 0 | 0 | 54 | 1.9 | 69/31 |  | Keep | Fulfil criteria |
| 10 | Has pain interfered with your daily activities? | 36 | 21 (58) | 0/1 | 2 | 0 | 54 | 2.4 | 48/52 |  | Keep | Fulfil criteria |
| 11 | Have you had headaches? | 38 | 20 (53) | 0/0 | 0 | 0 | 54 | 2.4 | 54/46 |  | Keep | Fulfil criteria |
| 12 | Have you been short of breath? | 27 | 19 (70) | 1/0 | 2 | 2 | 53 | 2.0 | 68/32 | Q 13 | Keep unchanged | Fulfil criteria  Different concepts |
| 13 | Have you had a feeling of tightness in your chest? | 24 | 15 (63) | 0/0 | 2 | 0 | 54 | 1.8 | 72/28 | Q 12  Q 14 | Keep unchanged | Fulfil criteria  Different concepts |
| 14 | Have you had pain in your chest? | 18 | 13 (72) | 0/0 | 2 | 0 | 54 | 1.6 | 76/24 | Q13 | Keep unchanged | Fulfil criteria  Different concepts |
| 15 | Have you coughed? | 44 | 23 (52) | 0/1 | 0 | 0 | 54 | 2.6 | 44/56 |  | Keep | Fulfil criteria |
| 16 | Have you coughed up phlegm? | 26 | 10 (38) | 0/0 | 0 | 0 | 54 | 1.8 | 78/22 |  | Keep | Fulfil criteria |
| 17 | Have you coughed up blood? | 4 | 2 (50) | 0/0 | 0 | 0 | 54 | **1.1** | **96/4** |  | Keep | Discussed, serious and clinically important |
| 18 | Have you had sticky saliva? | 16 | 5 (31) | 0/0 | 2 | 0 | 54 | 1.5 | 85/15 |  | Keep | Fulfil criteria |
| 19 | Have you had a sore throat? | 28 | 11 (39) | 0/0 | 2 | 0 | 54 | 1.8 | 76/24 |  | Keep | Fulfil criteria |
| 20 | Have you had feeling of tightness in your throat? | 18 | 11 (61) | 2/0 | 0 | 0 | 52 | 1.6 | 77/23 |  | Keep | Fulfil criteria |
| 21 | Have you had palpitations (faster or irregular heartbeat)? | 18 | 7 (39) | 0/0 | 0 | 0 | 54 | 1.6 | 85/15 |  | Keep | Fulfil criteria |
| 22 | Have you had a blocked nose? | 31 | **6 (19)** | 7/0 | 0 | 0 | 47 | 2.1 | 68/32 |  | Remove | Does not fulfil criteria 2. Not included in the list of problems long COVID |
| 23 | Have you been sneezing? | 29 | **4 (14)** | 6/0 | 0 | 0 | 48 | 1.9 | 79/21 |  | Remove | Does not fulfil criteria 2. Not included in the list of problems long COVID |
| 24 | Have you had aches or pains in your muscles or joints? | 36 | 22 (61) | 0/0 | 0 | 0 | 54 | 2.6 | 50/50 |  | Keep | Fulfil criteria |
| 25 | Have you had pain in your back? | 33 | 17 (52) | 1/0 | 0 | 0 | 53 | 2.3 | 62/38 |  | Keep | Fulfil criteria |
| 26 | Have you had stiffness in your muscles or joints? | 28 | 15(54) | 0/0 | 0 | 0 | 54 | 1.9 | 70/30 |  | Keep | Fulfil criteria |
| 27 | Have you had red eyes? | 15 | 7 (47) | 1/0 | 0 | 0 | 53 | 1.5 | 85/15 |  | Keep | Fulfil criteria |
| 28 | Have you had burning or sore eyes? | 18 | 7 (39) | 0/0 | 0 | 0 | 54 | 1.5 | **91/9** |  | Keep | Discussed, fulfil 5/6 criteria, decided to keep |
| 29 | Have you experienced reduced vision? | 11 | 5 (45) | 0/0 | 0 | 0 | 54 | **1.3** | **93/7** |  | Keep | Discussed, Fulfil 4/6 criteria  decided to keep only item on this issue |
| 30 | Has your sense of taste changed? | 27 | 16 (59) | 0/0 | 2 | 0 | 54 | 2.3 | 57/43 |  | Keep, change wording | Discussed proposal from patient, decided to include the direction of change |
| 30  New | *Have you had problems with your sense of taste?* |  |  |  |  |  |  |  |  |  |  |  |
| 31 | Has your sense of smell changed? | 27 | 17 (63) | 0/0 | 2 | 0 | 54 | 2.1 | 61/39 |  | Keep, change wording | Discussed proposal from patient, decided to include the direction of change |
| New | *Have you had problems with your sense of smell* |  |  |  |  |  |  |  |  |  |  |  |
| 32 | Have you had problems with hearing? | 9 | 6 (67) | 0/0 | 0 | 0 | 54 | **1.3** | **93/7** |  | Keep | Discussed, Fulfil 4/6 criteria  decided to keep only item on this issue |
| 33 | Have you had shooting or burning pain in your body? | 17 | 12 (71) | 0/0 | 2 | 0 | 54 | 1.6 | 81/19 |  | Keep, wording unchanged | Discussed wording, whether to include nerve pain or split in two Q. Clinically, shooting and burning cover neurologic pain, finally decided to keep unchanged |
| 34 | Have your hands been shaking? | 13 | **2 (15)** | 5/0 | 0 | 0 | 49 | **1.3** | **94/6** |  | Remove | Does not fulfil criteria 2. Also, not criteria 5 and 6 |
| 35 | Have you had weakness in your hands or feet? | 20 | 8 (40) | 2/0 | 0 | 0 | 52 | 1.7 | 81/19 |  | Keep | Fulfil criteria |
| 36 | Have you had tingling or numbness in your hands or feet? | 17 | 12 (71) | 0/0 | 0 | 0 | 54 | 1.6 | 78/22 |  | Keep | Fulfil criteria |
| 37 | Have you lacked appetite? | 35 | 14 (40) | 1/0 | 2 | 0 | 53 | 2.3 | 57/43 |  | Keep | Fulfil criteria |
| 38 | Have you had abdominal pain? | 22 | 10 (45) | 0/0 | 0 | 0 | 54 | 1.7 | 76/24 | Q39 | Keep unchanged | Overlap discussed, patients prefer Q39 to be removed |
| 39 | Have you had abdominal discomfort? | 21 | 9 (43) | 5/0 | 4 | 0 | 49 | 1.7 | 76/24 | Q38 | Remove | Overlap discussed, patients prefer Q39 to be removed |
| 40 | Have you had heartburn? | 15 | **2 (13)** | 5/0 | 0 | 0 | 49 | **1.4** | **92/8** |  | Remove | Does not fulfil criteria 2. Also, not criteria 5 and 6 |
| 41 | Have you felt nauseous? | 28 | 11 (39) | 0/0 | 0 | 0 | 54 | 1.8 | 81/19 |  | Keep | Fulfil criteria |
| 42 | Have you vomited? | 8 | 4 (50) | 0/0 | 0 | 0 | 54 | **1.3** | **93/7** |  | Keep | Discussed, does not fulfil criteria 5 and 6, but clinically important in acute phase. |
| 43 | Have you had diarrhoea? | 20 | 11 (54) | 0/0 | 0 | 0 | 54 | 1.7 | 78/22 |  | Keep | Fulfil criteria |
| 44 | Have you been constipated? | 8 | 4 (50) | 5/0 | 0 | 0 | 49 | **1.2** | **94/6** |  | Remove | Discussed. Does not fulfil crit 5 and 6. Can be caused by drugs? Decided to remove |
| 45 | Have you had pain or a burning feeling when passing urine? | 7 | 2 (29) | 5/0 | 0 | 0 | 49 | **1.2** | **94/6** |  | Remove |  |
| 46 | Have you had skin problems (e.g. itchy, dry, rash)? | 15 | 11 (73) | 0/0 | 0 | 0 | 54 | **1.4** | **93/7** |  |  | Discussed, Fulfil 4/6 criteria  decided to keep only item on this issue |
| 47 | Have you lost hair? | 3 | 2 (67) | 5/0 | 0 | 0 | 49 | **1.1** | **96/4** |  | Remove | Discussed in the group. Does not fulfil criteria 5 and 6. Majority in favour of removing |
| 48 | Have you felt anxious? | 36 | 22 (61) | 0/0 | 2 | 0 | 54 | 2.4 | 59/41 | Q51,  Q52 | Keep | Different concept from Q52. Discussed whether to remove Q51 based on patients feedback |
| 49 | Have you felt sad? | 21 | 18 (86) | 0/0 | 2 | 2 | 54 | 2.1 | 69/31 |  | Keep |  |
| 50 | Have you felt depressed? | 24 | 12 (50) | 0/0 | 0 | 0 | 54 | **1.8** | 80/20 |  | Keep | Fulfil 5/6 criteria, decided to keep |
| 51 | Have you felt tense? | 30 | 21 (70) | 0/0 | 0 | 0 | 54 | 2.1 | 63/37 | Q48, Q52 | Keep | Different meaning in different languages. Important to keep for India – interpreted as worried |
| 52 | Have you felt restless or agitated? | 29 | 14 (48) | 0/0 | 0 | 0 | 54 | 1.9 | 78/22 | Q48, Q51 | Keep | Different concept from Q48 |
| 53 | Have you felt angry (with yourself or others) for getting the virus? | 20 | 9 (45) | 0/0 | 2 | 0 | 54 | 1.6 | 81/19 |  | Keep |  |
| 54 | Have you had upsetting dreams? | 12 | 4 (33) | 0/0 | 0 | 0 | 54 | **1.4** | 89/11 |  | Keep | Fulfil 5/6 criteria, decided to keep |
| 55 | Have you felt lonely? | 19 | 7 (37) | 0/0 | 0 | 0 | 54 | 1.6 | 85/15 |  | Keep | Fulfil all criteria |
| 56 | Have you felt unable to cope mentally with the situation caused by your disease? | 18 | 12 (67) | 0/0 | 0 | 0 | 54 | 1.6 | 83/17 |  | Keep | Fulfil all criteria |
| 57 | Have you felt confused? | 18 | 8 (44) | 0/0 | 0 | 0 | 54 | 1.6 | 81/19 |  | Keep | Fulfil all criteria |
| 58 | Have you had problems maintaining concentration even when doing something important? | 21 | 12 (57) | 0/0 | 0 | 0 | 54 | 1.6 | 83/17 |  | Keep | Fulfil all criteria |
| 59 | Have you had problems remembering things from the last couple of days? | 18 | 10 (56) | 0/0 | 2 | 0 | 54 | 1.5 | 85/15 |  | Keep | Fulfil all criteria |
| 60 | Have you had problems remembering things from the time before your infection? | 8 | 4 (50) | 0/0 | 2 | 0 | 54 | **1.2** | **94/6** |  | Keep | Discussed, important for cognitive function and long COVID |
| 61 | If you tried, would you have problems carrying a heavy bag upstairs? | 31 | 14 (45) | 0/0 | 0 | 0 | 54 | 2.2 | 63/37 |  | Keep | Fulfil all criteria |
| 62 | If you tried, would you have problems walking 100 m? | 27 | 12 (44) | 1/0 | 2 | 0 | 53 | 2.0 | 70/30 |  | Keep | Fulfil all criteria |
| 63 | Have you needed help dressing? | 9 | 6 (67) | 0/0 | 0 | 0 | 54 | **1.3** | 89/11 |  | Keep | Discussed, fulfil 5/6 criteria important to assess different levels of physical functioning |
| 64 | Have you been limited in doing heavy housework? | 33 | 15 (45) | 0/0 | 2 | 0 | 54 | 2.5 | 50/50 |  | Keep | Fulfil all criteria |
| 65 | Have you been limited in doing light housework? | 27 | 16 (59) | 1/0 | 2 | 0 | 53 | 2.2 | 55/45 |  | Keep | Fulfil all criteria |
| 66 | Have you been limited in doing either your work or other daily activities? | 35 | 22 (63) | 1/0 | 0 | 0 | 53 | 2.5 | 45/55 |  | Keep | Fulfil all criteria |
| 67 | Has your physical condition or medical treatment interfered with your social activities? | 40 | 22 (55) | 0/0 | 0 | 0 | 54 | 2.9 | 37/63 |  | Keep | Fulfil all criteria |
| 68 | As a result of your physical condition or medical treatment, have you felt isolated from your family or friends? | 32 | 21 (66) | 0/0 | 2 | 0 | 54 | 2.3 | 59/41 |  | Keep | Fulfil all criteria |
| 69 | Have you worried about infecting others with the virus? | 39 | 33 (85) | 0/0 | 0 | 0 | 54 | 2.9 | 32/68 |  | Keep | Fulfil all criteria |
| 70 | Have you felt guilty or ashamed because you might have infected others with the virus | 33 | 21 (64) | 0/0 | 0 | 0 | 54 | 2.2 | 59/41 |  | Keep | Fulfil all criteria |
| 71 | Have you worried that you might be stigmatised because of your illness? | 21 | 8 (38) | 0/0 | **9** | 2 | 54 | 1.7 | 78/22 |  | Keep, change wording | Fulfil all criteria, “stigmatised” were difficult to understand in some languages. Decided to include “or judged negatively” |
| 71 New | *Have you worried that you might be stigmatised or judged negatively because of your illness?* |  |  |  |  |  |  |  |  |  |  |  |
| 72 | Have you worried about your health in the future? | 43 | 25 (58) | 0/0 | 0 | 0 | 54 | 2.6 | 43/57 |  | Keep | Fulfil all criteria |
| 73 | Have you worried about your physical condition or medical treatment causing you financial difficulties? | 21 | 12 (57) | 0/0 | 0 | 0 | 54 | 1.8 | 76/24 |  | Keep | Fulfil all criteria |
| 74 | Have you worried that you might not get support from family or friends? | 7 | 5 (71) | 0/0 | 0 | 2 | 54 | **1.3** | **93/7** | Q76 | Keep | Discussed, decided to keep 74, remove Q76. Discussed if “emotional” should be added, but wanted to cover also practical support |
| 75 | Have you worried about being isolated from those close to you (e.g. family, friends)? | 28 | 12 (43) | 1/0 | 0 | 0 | 54 | 2.0 | 72/28 |  | Keep | Fulfil all criteria |
| 76 | Have you worried about being abandoned by family or friends? | 7 | 7 (100) | 5/0 | 0 | 0 | 54 | **1.2** | **96/4** |  | Remove | Discussed, decided to keep 74, remove Q76 |
| 77 | Have you worried about being abandoned by health care professionals? | 6 | 3 (50) | 0/0 | 0 | 0 | 54 | **1.2** | **91/9** |  | Keep, change wording | Fulfil 4/6 criteria. Only item on health care professionals and included by patients in phase I.  Wording: Abandoned is to offensive in some languages Patients who introduced this in phase I experienced being left all alone in the room without help |
| 77  New | *Have you worried that you might not receive sufficient attention from health care professionals* |  |  |  |  |  |  |  |  |  |  |  |
| 78 | Have you had problems communicating with health care professionals due to personal protective equipment (e.g. wearing masks)? | 9 | 4 (44) | 0/1 | 0 | 0 | 54 | **1.2** | 96/4 |  | Keep | Fulfil 5/6 criteria, decided to keep |
| 79 | How would you rate your overall health during the past week?* | 39 | 24 (62) | 14/2 | 2 | 2 | 54 | 3.3 | 20/80 |  |  | Fulfil criteria, high proportion of missing on relevance, due to score 1-4 not suitable |
| 80 | How would you rate your overall quality of life during the past week?* | 40 | 29 (73) | 13/1 | 2 | 0 | 54 | 3.3 | 22/88 |  |  | Fulfil criteria, high proportion of missing on relevance, due to score 1-4 not suitable |

**In bold:** criterion for removal met

Highlighted light grey: candidates for removal, discussed at the meeting

Highlighted in darker grey: final decision to remove the item

*In italic: item rephrased*

Question (Q)

**Appendix 9 Distribution of patients per country (n = 371)**

|  | At home | In hospital or nursing home |
| --- | --- | --- |
| Austria - German | 4 | 12 |
| Croatia - Croatian | 18 | 12 |
| Germany - German | 36 | 10 |
| Ghana - Twi | 0 | 15 |
| Ghana- English | 14 | 14 |
| India - Gujarati | 45 | 0 |
| Norway - Norwegian | 32 | 12 |
| Palestine - Arabic | 13 | 25             + 1 unspecified |
| Spain - Spanish | 34 | 6 |
| Sweden - Swedish | 36 | 1 |
| United Kingdom - English | 31 | 0 |

**Appendix 10: Patient-reported additional issues**

| **Issue** | **Covered by item** | **Short term**  **n = 32** | **Long term**  **n = 21** |
| --- | --- | --- | --- |
| Menstrual disturbances | New | 1 | 1 |
| Aphasia/wordfinding problems | New | 1 | 1 |
| Fatigue | Q3,Q4-Q5 | 16 | 10 |
| Tiredness/exhaustion | Q5 | 5 | 2 |
| Insomnia | Q7 | 4 | 3 |
| Dizziness | Q9 | 2 | 1 |
| Headache | Q11 | 3 | 2 |
| Dyspnea/shortness of breath | Q12 | 9 | 3 |
| Chest tightness/high congestion | Q13? | 1 | 1 |
| Cough | Q15 | 1 |  |
| Productive cough | Q16 | 1 |  |
| Cardiac flushing/heart palpitations | Q21 | 4 | 2 |
| Body pain | Q10, Q22,Q23 | 1 | 1 |
| Muscle soreness/muscle and joint pain | Q22 | 7 | 5 |
| Back pain | Q23 | 1 |  |
| Muscle stiffness | Q24 | 1 |  |
| Eye pressure/eye problems | Q25,Q26 | 1 |  |
| Loss of taste | Q28 | 4 | 3 |
| Loss of smell | Q29 | 9 | 6 |
| Shooting or burning pain in your body | Q31 |  | 1 |
| Tingling hands and feet | Q33 | 2 | 2 |
| Vomiting | Q37 | 1 | 1 |
| Diarrhea | Q38 | 1 |  |
| Skin burning | Q39 | 1 |  |
| Anxiety | Q40 | 2 | 3 |
| Depressed | Q42 | 1 |  |
| Retentiveness | Q43 | 1 | 1 |
| Restless/irritable | Q44 | 2 |  |
| Distorted perception | Q48-49/ Q33 | 2 |  |
| Cognitive/difficult focusing/concentrating | Q48-52 | 6 | 6 |
| Memory loss/problems | Q51, Q52 | 4 | 3 |
| Low performance | Q56-57 | 1 | 1 |
| Became weaker due to isolation | Q58-60 | 2 |  |
| Worry that will be unable to socialize due to tiredness | Q59 | 1 |  |
| Isolated from family led to worry and anxiety | Q66-67 | 1 |  |
| That acquaintances cannot accept that various symptoms, can be after-effects of Corona | Q63 |  | 1 |
|  | **Excluded previously** |  |  |
| Feel cold | Phase I | 1 | 1 |
| Tinnitus | Phase I | 2 | 2 |
| Nasal secretion | Phase I | 3 |  |
| Trembling | Phase I | 1 |  |
| Hearing loss | Phase I |  | 1 |
| Panic attack | Phase I |  | 1 |
| Nose congestion | Phase IIIA | 1 |  |
| Congestion (constipation) | Phase IIIA | 1 |  |
| Loss of hair | Phase IIIA | 3 | 1 |
| Sweating | Phase IIIA | 1 |  |
| Dysuria | Phase IIIA | 1 |  |
|  | **No symptom** |  |  |
| Aneurism | X | 1 |  |
| Heart/lung | X | 3 | 2 |
| Throat and lung infection | X | 2 |  |
| Low oxygen saturation | X | 1 |  |
| Thrombophlebitis | X | 1 |  |
| Syncope | X, discussed previously no symptom, covered by Q8-9 | 1 | 1 |
| Bone edema in the femur | X |  | 1 |
| Deep venous thrombosis | X |  | 1 |
| Weight loss | X |  | 1 |
| Hypertension | X |  | 1 |
| Psoriasis | X |  | 1 |
