# Supplementary material for: A multicenter international prospective study of the validity and reliability of a COVID-19-specific health-related quality of life questionnaire
Source: Qual Life Res. 2022 Oct 23;32(2):447–59. doi: 10.1007/s11136-022-03272-2 (PMC9589865; doi:10.1007/s11136-022-03272-2)
Supplement: Supplementary file 3 — Supplementary file3 (DOCX 45 kb) [file 11136_2022_3272_MOESM3_ESM.docx]

**An international developed health-related quality of life questionnaire for patients with COVID-19**

**Scoring manual for the Oslo COVID-19 QLQ-W61©**

**Format:** The Oslo COVID-19 QLQ-W61© is a 61-item self-reporting quality of life questionnaire

**Target group:** Patients with COVID-19, at diagnosis, during active disease and in the early and late recover phase.

**Level of development:** The current version has been tested and preliminary validated in phase III and has a one week recall-period (W).

**Content:** It is composed of 15 multi-item scales and six single item measures. The multi-item scales include from two to eight different items. No item occur in more than one scale.

**History of development:** The current version, The Oslo COVID-19 QLQ-W61© was developed according to international guidelines and the development process has been published (1, 2).

**The next generation:** The questionnaire has been tested in a larger international sample of patients, and the results from the analyses will be published in the near future. Further validation of the scale structure and sensitivity to change will be performed in phase IV.

**Table 1: Overview of the Oslo COVID-19 QLQ-W61© items and scales**

| **#** | **Item** | **Scale number** | **Scale name** |
| --- | --- | --- | --- |
| 1 | Have you had fevers? | 1 | Temperature disruption |
| 2 | Have you had chills? | 1 | Temperature disruption |
| 3 | Have you needed to rest? | 2 | Fatigue |
| 4 | Have you felt weak? | 2 | Fatigue |
| 5 | Have you been tired? | 2 | Fatigue |
| 6 | Have you felt drowsy? | 4 | Malaise |
| 7 | Have you had problems sleeping? | 3 | Sleep - Single item |
| 8 | Have you felt ill or unwell? | 4 | Malaise |
| 9 | Have you been dizzy? | 4 | Malaise |
| 10 | Has pain interfered with your daily activities? | 5 | Pain |
| 11 | Have you had headaches? | 3 | Malaise |
| 12 | Have you been short of breath? | 6 | Respiratory lower, chest |
| 13 | Have you had a feeling of tightness in your chest? | 6 | Respiratory lower, chest |
| 14 | Have you had pain in your chest? | 6 | Respiratory lower, chest |
| 15 | Have you coughed? | 6 | Respiratory lower, chest |
| 16 | Have you coughed up phlegm? | 6 | Respiratory lower, chest |
| 17 | Have you had sticky saliva? | 7 | Respiratory upper, throat |
| 18 | Have you had a sore throat? | 7 | Respiratory upper, throat |
| 19 | Have you had feeling of tightness in your throat? | 7 | Respiratory upper, throat |
| 20 | Have you had palpitations (faster or irregular heartbeat)? | 8 | Palpitations - single item |
| 21 | Have you had aches or pains in your muscles or joints? | 5 | Pain |
| 22 | Have you had pain in your back? | 5 | Pain |
| 23 | Have you had stiffness in your muscles or joints? | 5 | Pain |
| 24 | Have you had burning or sore eyes? | 9 | Eye - Single item |
| 25 | Have you had problems with your sense of taste? | 10 | Sensory |
| 26 | Have you had problems with your sense of smell | 10 | Sensory |
| 27 | Have you had shooting or burning pain in your body? | 5 | Pain |
| 28 | Have you had weakness in your hands or feet? | 11 | Neurological |
| 29 | Have you had tingling or numbness in your hands or feet? | 11 | Neurological |
| 30 | Have you lacked appetite? | 12 | Appetite loss - Single item |
| 31 | Have you had abdominal pain? | 13 | Gastrointestinal |
| 32 | Have you felt nauseous? | 13 | Gastrointestinal |
| 33 | Have you had diarrhoea? | 13 | Gastrointestinal |
| 34 | Have you had skin problems (e.g. itchy, dry, rash)? | 14 | Skin - Single item |
| 35 | Have you felt anxious? | 15 | Emotional functioning |
| 36 | Have you felt sad? | 15 | Emotional functioning |
| 37 | Have you felt depressed? | 15 | Emotional functioning |
| 38 | Have you felt tense? | 15 | Emotional functioning |
| 39 | Have you felt restless or agitated? | 15 | Emotional functioning |
| 40 | Have you felt angry (with yourself or others) for getting ill? | 15 | Emotional functioning |
| 41 | Have you had upsetting dreams? | 15 | Emotional functioning |
| 42 | Have you felt lonely? | 15 | Emotional functioning |
| 43 | Have you had problems maintaining concentration even when doing something important? | 16 | Cognitive functioning |
| 44 | Have you had problems remembering things from the last couple of days? | 16 | Cognitive functioning |
| 45 | Have you had problems remembering things from the time before your infection? | 16 | Cognitive functioning |
| 46 | If you tried, would you have problems carrying a heavy bag upstairs? | 17 | Physical functioning |
| 47 | If you tried, would you have problems walking 100 m? | 17 | Physical functioning |
| 48 | Have you needed help dressing? | 17 | Physical functioning |
| 49 | Have you been limited in doing either your work or other daily activities? | 18 | Role functioning - Single item |
| 50 | Has your physical condition or medical treatment interfered with your social activities? | 19 | Social functioning |
| 51 | As a result of your physical condition or medical treatment, have you felt isolated from your family or friends? | 19 | Social functioning |
| 52 | Have you worried about infecting others with the virus? | 20 | Worries |
| 53 | Have you felt guilty or ashamed because you might have infected others with the virus | 20 | Worries |
| 54 | Have you worried that you might be stigmatised or judged negatively because of your illness? | 20 | Worries |
| 55 | Have you worried about your health in the future? | 20 | Worries |
| 56 | Have you worried about your physical condition or medical treatment causing you financial difficulties? | 20 | Worries |
| 57 | Have you worried that you might not get support from family or friends? | 20 | Worries |
| 58 | Have you worried about being isolated from those close to you (e.g. family, friends)? | 20 | Worries |
| 59 | Have you worried that you might not receive sufficient attention from health care professionals | 20 | Worries |
| 60 | How would you rate your overall health during the past week? | 21 | Overall quality of life |
| 61 | How would you rate your overall quality of life during the past week? | 21 | Overall quality of life |

**Multi-item and single item scales within the questionnaire:** In the Oslo COVID-19 QLQ-W61, 15 multi-item scales (table 2) and six single item scales (table 3) have been constructed based upon the analyses of the data from international patient sample (ref Amdal et al, Pretesting and validation of an international COVID-19 specific health-related quality of life questionnaire, publication to be submitted).

**Table 2: The Oslo COVID-19 QLQ-W61© multi-item scales (n = 15)**

| **Scale name** | **Acronym** | **Question number** |
| --- | --- | --- |
| Temperature disruption | TP | q1, q2 |
| Fatigue | FA | q3, q4, q5 |
| Malaise | MA | q6, q8, q9, q11 |
| Respiratory lower, chest | RL | q12, q13, q14, q15, q16 |
| Respiratory upper, throat | RU | q17, q18, q19 |
| Pain | PA | q10, q21, q22, q23, q27 |
| Sensory | SE | q25, q26 |
| Neurological | NE | q28, q29 |
| Gastrointestinal | GI | q31, q32, q33 |
| Emotional functioning | EF | q35, q36, q37, q38, q39, q40, q41, q42 |
| Cognitive functioning | CF | q43, q44, q45 |
| Physical functioning | PF | q46, q47, q48 |
| Social functioning | SF | q50, q51 |
| Worries | WO | q52, q53, q54, q55, q56, q57, q58, q59 |
| Overall quality of life | QOL | q60, q61 |

**Table 3: The Oslo COVID-19 QLQ-W61©** **single item scales (n = 6)**

| **Name** | **Acronym** | **Question number** |
| --- | --- | --- |
| Sleep | SL | q7 |
| Palpitation | PP | q20 |
| Eye | EY | q24 |
| Appetite loss | AP | q30 |
| Skin | SK | q34 |
| Role functioning | SF | q49 |

**Scoring of scales and single items**: All items are scored on a four-point Likert scale ranging from “not at all” (1) to “very much” (4), except for the overall quality of life scale (items 60 and 61) ranging from “very poor” (1) to “excellent” (7).

The scales and single items (except overall quality of life) measure the degree of symptoms or limitation in functioning. The overall quality of life scale (q60, q61) measures a positive outcome.

The raw scores from all scales and single items are linearly transformed from the raw scores (1 - 4 or 1 – 7) to scores so that scores range from 0 to 100; a higher score represents higher (worse) level of symptoms and more limited (worse) functioning. For the overall quality of life scale, higher score represents better quality of life. This allows all scales and single items (except overall quality of life) to have the same direction in presentations in accordance with international recommendations (3).

| **Technical Summary** (A Syntax Stata will be provided with variable names and labels)  The principle of the scoring of the scales is the same in all cases, for multi-item scales and single items:   1. Estimate the average score of the item(s) that contribute to the scale i.e. sum the included item scores and divide by the number of responded items; this is the **raw score**.      1. Use a linear transformation to standardize the raw score, so that scores range from 0 to 100 by the formula scale score = (raw score-1)/(4-1) x 100. 2. For overall quality of life {1- (raw score-1)/(7-1)} x 100. |
| --- |

**Report of missing items and missing forms/questionnaires:** The scoring algorithm above can be utilized if half or more of the items within each scale are completed. Otherwise, the scale is set as missing. It is not acceptable to include any of the single items into the multi-item scales as this would hamper the validity of the results from the questionnaire.

According to the CONSORT PRO extension (4), it is important reporting the fraction/ proportion of patients in the study that completed the questionnaire and to report the number of missing values.

**Clinical important difference or change:** The threshold value for clinical important difference and clinical important change for the OSLO COVID-19 QLQ-W61© has not yet been determined.

As the structure and scoring of the COVID-19 QLQ-W61© is based on the structure and scoring of the EORTC questionnaires one could consider using the same rule of thumb with a clinically important difference defined as a difference of 10 in absolute scores based on the publication by Osoba and co-workers (5).

**Presentation/ publication of results:** Ideally, before the start of the data analyses, select the subscales of special interest and present the results accordingly together with other clinical results in the main paper.

In order to capture a broader diversity of the patients’ situation, we strongly recommend publishing the results from all subscales and single items in a descriptive manner as tables/supplementary tables.

To improve the understandability for clinicians and patients, we recommend presentation of fractions/proportion of patients with problems/symptoms. Please review other guidelines on presentation of PROM results recommend using Bar charts with confidence intervals to make it easier to read (Claire Snyder 2018 (3)).

Any publication that describes the use of the OSLO COVID-19 QLQ-W61©, or analyses of data arising from this questionnaire should explicitly cite the following references (1, 2):

1. Amdal CD, Pe M, Falk RS, Piccinin C, Bottomley A, Arraras JI, Darlington AS, Hofsø K, Holzner B, Jørgensen NMH, Kulis D, Rimehaug SA, Singer S, Taylor K, Wheelwright S, Bjordal K. Health-related quality of life issues, including symptoms, in patients with active COVID-19 or post COVID-19; a systematic literature review.
   Qual Life Res. 2021 Dec;30(12):3367-3381. doi: 10.1007/s11136-021-02908-z. Epub 2021 Jun 19.
2. Cecilie Delphin Amdal; Katherine Taylor; Dagmara Kuliś; Ragnhild Sørum Falk; Andrew Bottomley; Juan Ignacio Arraras; James Harold Barte; Anne Sophie Darlington; Kristin Hofsø; Bernard Holzner; Nina Marie Høyning Jørgensen; Melissa Paulita Mariano; Madeline Pe; Claire Piccinin; Nicola Riccetti; Melanie Schranz; Sally Wheelwright; Kristin Bjordal. Health-related quality of life in patients with COVID-19; international development of a patient-reported outcome measure.
   Accepted for publication in the Journal of Patient-Reported Outcomes, 15 February 2022.

**References**

1. Amdal CD, Pe M, Falk RS, Piccinin C, Bottomley A, Arraras JI, et al. Health-related quality of life issues, including symptoms, in patients with active COVID-19 or post COVID-19; a systematic literature review. Qual Life Res. 2021:1-15.

2. Amdal CD TK, Kuliś D, Falk RS, Bottomley A, Arraras JI, et al. Health-related quality of life in patients with COVID-19; international development of a patient-reported outcome Submittet for publication2022.

3. Snyder C, Smith K, Holzner B, Rivera YM, Bantug E, Brundage M. Making a picture worth a thousand numbers: recommendations for graphically displaying patient-reported outcomes data. Qual Life Res. 2019;28(2):345-56.

4. Calvert M, Blazeby J, Altman DG, Revicki DA, Moher D, Brundage MD. Reporting of patient-reported outcomes in randomized trials: the CONSORT PRO extension. JAMA. 2013;309(8):814-22.

5. Osoba D. Current applications of health-related quality-of-life assessment in oncology. Support Care Cancer. 1997;5(2):100-4.
